# A FRAUD AWARENESS MAIL CAMPAIGN SIGNIFICANTLY REDUCES RATES OF REVICTIMIZATION AMONG OLDER VICTIMS

**DOI:** 10.1093/geroni/igae098.0367

**Published:** 2024-12-31

**Authors:** Marguerite DeLiema, M Daniel Brannock, Edward Preble, Lynn Langton

**Affiliations:** University of Minnesota Twin Cities, St. Paul, Minnesota, United States; RTI International, Research Triangle Park, North Carolina, United States; RTI International, Research Triangle Park, North Carolina, United States; RTI International, Research Triangle Park, North Carolina, United States

## Abstract

Older adults are disproportionately targeted by scams, with direct cost estimates ranging from $5.9 to 48.4 billion per year (Federal Trade Commission, 2023). Much of these costs are driven by repeat and chronic victimization. We conducted a randomized control trial to evaluate the efficacy of a mailed intervention to prevent revictimization among known older adult mail fraud victims. Participants’ addresses were randomly assigned to one of two treatment groups or to a control. Treatment groups received either (1) a single letter from the US Postal Inspection Service (USPIS) informing them that they paid money in response to a mail scam and may be targeted by additional scams, as well as education on how to spot mail scams, or (2) the same letter along with five additional “Fraud Fighter” awareness materials mailed two weeks apart. The control group received no materials. The effectiveness of the intervention was assessed through actual victim behavior. USPIS collected return addresses from detained victim mail for 4 months following the initial intervention mailing. We identified revictimization by matching the return addresses from the newly detained victim mail to the addresses of victims in the experiment. Compared to the control (no intervention), the single letter condition reduced the rate of revictimization by 8.6% and the multiple mailing condition reduced the rate of revictimization by 22.4% over 4 months. This represents the first test of a simple, scalable, mailed intervention to reduce the incidence and cost of mass marketing fraud among older Americans.

